# Improvement of the mechanical property and thermal stability of polypropylene/recycled rubber composite by chemical modification and physical blending

**DOI:** 10.1038/s41598-020-59191-0

**Published:** 2020-02-12

**Authors:** Tai-Chin Chiang, Huan-Li Liu, Lung-Chang Tsai, Tao Jiang, Ning Ma, Fang-Chang Tsai

**Affiliations:** 1 Office of Research and Development, National Taiwan University of Science and Technology, 10607 Taipei, Taiwan; 2 Hubei Key Laboratory of Polymer Materials, Key Laboratory for the Green Preparation and Application of Functional Materials (Ministry of Education), Hubei Collaborative Innovation Center for Advanced Organic Chemical Materials, School of Materials Science and Engineering, Hubei University, Wuhan, 430062 China; 3 College of Chemistry and Molecular Engineering, Peking University, Beijing National Laboratory for Molecular Sciences (BNLMS), Beijing, 100871 People’s Republic of China; 4 College of Biology and Agricultural Resources, Huanggang Normal University, Huanggang, 438000 People’s Republic of China

**Keywords:** Structural materials, Environmental impact

## Abstract

The binary blend materials containing the modified recycled rubber powder with maleic a hydride modified polypropylene thermoplastic elastomer were prepared by dynamic vulcanization and blended with a variety of additives such as activated agent, accelerator, solubilizer, and the crosslinking agent. The thermal properties and mechanical properties including tensile strengths and impact strengths of pristine rubber, polypropylene and their corresponding binary blends were investigated. Besides, the effects of the amount of rubber powder, polypropylene, crosslinking agent, accelerator, activator, and solvent were studied and the microstructures of the pristine rubber, pristine polypropylene, and their corresponding binary blends were observed by scanning electron microscopy. It was found that the compatibilizer could effectively disperse the size of 120 mesh of recycled rubber powder into the polypropylene in the same manner and the homogeneous tear section of the rubber/polypropylene thermoplastic elastomer was obtained. The results on the effects of additives on mechanical and morphological properties of recycled rubber/polypropylene binary blends guide the rational design of novel polymeric composites from recycled polymeric materials.

## Introduction

With the development of automobile industry, massive tire production have been dramatically generate a large amount (about 1.4 million tons) of waste rubber in the past few decades^1^. The most generic method to dispose the waste tires is stacking, landfilling, and incineration. While these “black garbage” brings environmental pollution even they are stacked, landfilled or incinerated. For example the piled tires occupy land resources leading to breed mosquitoes and spread diseases^2,3^. Therefore, recycling waste rubbers to reduce environmental pollution have attracted widespread attention. It should be meaningful to develop an environment-friendly approach for efficiently recycling even once of those rubbers^4^.

The most well-developed application of recycled scrap rubber is used in the direction of modified asphalt, which is mainly used for road paving^4^. One of the feasible methods such as a direct mixing of rubber powder/TPV (Thermoplastic Vulcanizate) blend materials was investigated and studied by the addition of compatibilizers to improve the comprehensive performance of blending materials^5^. It has been reported that the compatibility of epoxy resin and the colloidal powder was improved by the method of coating with a silane coupling agent, which can greatly improve the frangibility and thermal stability of epoxy resin^6^. Besides, the waste rubber powder/polypropylene blends have been prepared by using acrylamide to modify the waste rubber powder under a UV light irradiation. The tensile properties of the waste rubber powder/polypropylene were significantly improved compared to that of the unmodified systems. Li Yan *et al*. have reported that the mechanical properties of PVC (Polyvinyl Chloride)/rubber powder can be improved by using oxygen plasma to modify the surface of the waste materials^7^.

**Scheme 1:**
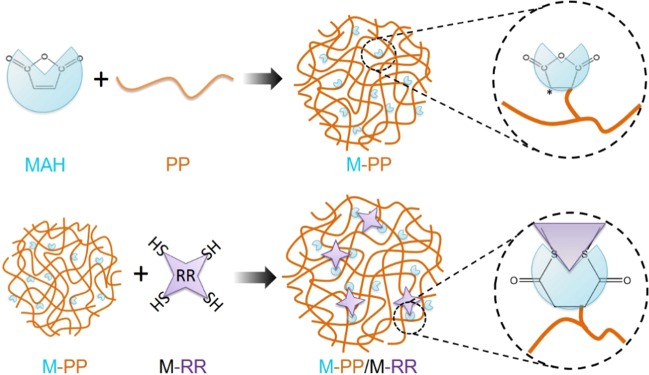
The preparation process of M-PP/M-RR. (Drawn by Ning Ma and Fang-Chang Tsai).

Herein, we report a chain rupture and rebinding way to reuse the waste rubber powders (Scheme 1). Compared to the previous work, recycled rubber powder in this study was pre-treated by desulfurized solution. Then, the modified waste rubber powders blends with MAH-polypropylene and the control samples were investigated by using thermal analysis, Fourier-transform infrared spectroscopy (FT-IR) analysis, mechanical and Scanning Electron Microscope (SEM) to explore a better combination of the recycled rubber powder/polypropylene binary blend systems. The interrelationship of the microstructures, the thermal and the mechanical properties of the pristine rubber, pristine polypropylene and their binary blends were also studied for future applications.

## Experimental

### Materials

Chemicals, reagents, and solvents including recycled rubber (RR) powder were supplied by Xiaogan Sanyang Plastics Technology Co., Ltd., Hubei, China (120 mesh), which mainly consist of butadiene rubber and carbon black. The available polypropylene (PP) with the trade name of T300 was supplied from SINOPEC Shanghai Petrochemical Co. Ltd., China, Maleic anhydride (MAH) (≥99.5% purity) was offered by Tianjin Bodi Chemical Industry Co. Ltd., China, Dicumyl peroxide (DCP, Tokyo Chemical Industry Co. Ltd., Japan), antioxidant 1010 (Shanghai Chic New Material Co. Ltd., China), toluene, FeCl_2_, acetic acid, and hydrochloric acid are obtained from commercial sources and used without further purification unless otherwise noted.

### Preparation of maleic anhydride-modified polypropylene (M-PP)

M-PP was synthesized by employing the previous reported paper^8,9^. A mixture of 95 g polypropylene (PP), 4 g maleic anhydride (MAH), 0.3 g dicumyl peroxide (DCP) and 0.6 g antioxidant 1010 was stirred thoroughly at room temperature for 10 min to mix thoroughly. The mixture was then added into a Micro Internal Mixer (SU-70 ML) at 180 °C for 20 min using the speed rate of 36 revolutions per minute (rpm). The M-PP was obtained and kept in the dryer for further use.

### Preparation of desulfurized solution

FeCl_2_ (4 g) was added in a mixture of toluene (11.5 mL), acetic acid (3.8 mL) and 30% of hydrochloric acid (1.7 mL) and stirred at the room temperature.

### Preparation of modified rubber (M-RR)

The recycled rubber powder (80 g) was mixed with the desulfurized solution at room temperature. The mixture was then added in the micromixer at 150 °C with a speed rate of 36 rpm and stirred for 20 min. The modified rubber was obtained and kept in the dryer for further use.

### Preparation of PP/RR

The ratio of the materials was studied by the relevant literature^10^. The recycled rubber (RR) and polypropylene (PP) in a percentage ratio of 70% to 30% were mixed with a total mass of 50 g. The mixture was then added in the micromixer and the final product was obtained as a back solid.

### Preparation of M-PP/RR

A similar procedure was used as that described for the preparation of M-PP/M-RR using M-PP (30%) and RR (70%) with a total mass of 50 g. The mixture was then added in the micromixer and a small amount of DCP was then added as a crosslinking agent at 150 °C with a speed rate of 36 rpm and stirred thoroughly for 7 min.

### Preparation of PP/M-RR

A similar procedure was used as that described for the preparation of M-PP/M-RR using M-PP (30%) and M-RR (70%) with a total mass of 50 g. The mixture was added in the micromixer with a small amount of the crosslinking agent (DCP) at the same procedure described for M-PP/RR.

### Preparation of M-PP/M-RR

The certain percentages of M-RR (70%) and M-PP (30%) were mixed with a total mass of 50 g. The mixture was then added in the micromixer and a small amount of DCP was then added as a crosslinking agent at 150 °C with a speed rate of 36 rpm and stirred thoroughly for 7 min.

### Sample handling

The M-PP/modified rubber was completely broken into granules by YL-2230 Pulverizer. The granules (3.5 g) were added in the preforming mold with a size of 10 × 10 × 0.5 cm^3^. The preforming process was implemented by a ZG-50 automatic pressure machine. Specific steps are as follows:

1. The sample was preheated for 10 min while maintaining the full contact of the pressure plate and the heating plate, and the pressure should maintain with no pressure;
2. To release the gas produced by heating, manually operation of the pressure plate on the pressure of 0 MPa and the maximum (30 MPa) for ten times is needed. The sample was then under the maximum pressure for 20 min;
3. The sample with performing mold was placed on the cold press plate of the QLB-350 × 350 × 2 plate vulcanization machines for 5 min.

### Tension splines handling

A dumbbell-like slicer was placed on the pressed sheet and then, CP-25 sheet-punching machine was used for tension splines molding (ASTM D638). Tension splines can be used for subsequent characterization and the experiments should be without any obvious edges, white, crack or other defects.

### Impact splines handling

The splines were pentad sandwiched between XQZH-2 type notches on the prototype of the jig. Then notch cutting makes the gap in V-shape (ASTM D256). Impact splines can be used for subsequent characterization and the experiments should be without any spline gap, obvious defects and evenly thickness of the spline.

### Characterization

Scanning Electron Microscopy (SEM) was carried out by a JSM-6510LV scanning electron microscopy (JEOL Ltd., Japan). The fracture surface sample was treated by liquid nitrogen and broken by hammer. Samples in KBr chips were analyzed by Fourier Transform-Infrared Spectroscopy (FT-IR) spectrophotometer (Nicolet iS50, Thermo Fisher Scientific Inc., USA). Thermal Gravimetric Analysis (TGA) was performed on a TGS-2 thermogravimetric analyzer (PerkinElmer, Inc., USA). Dynamic Thermomechanical Analysis (DMA) was carried out by a DMA Q800 V21.1 Build 51 Dynamic Mechanical Analyzer (TA Instruments, Inc., USA). The tensile strength and breaking elongation, the tensile strength and breaking elongation were carried out by a CMT-4104 Microcomputer control electronic universal testing machine (Shenzhen SANS Testing Machine Co., Ltd., Japan). The stretching rate was set at 20 mm/min until broken. The impact strength was carried out by a GT-7045-HML digital impact testing machine (Gotech Testing Machines Inc., Taiwan).

## Results and Discussion

**Figure 1:**
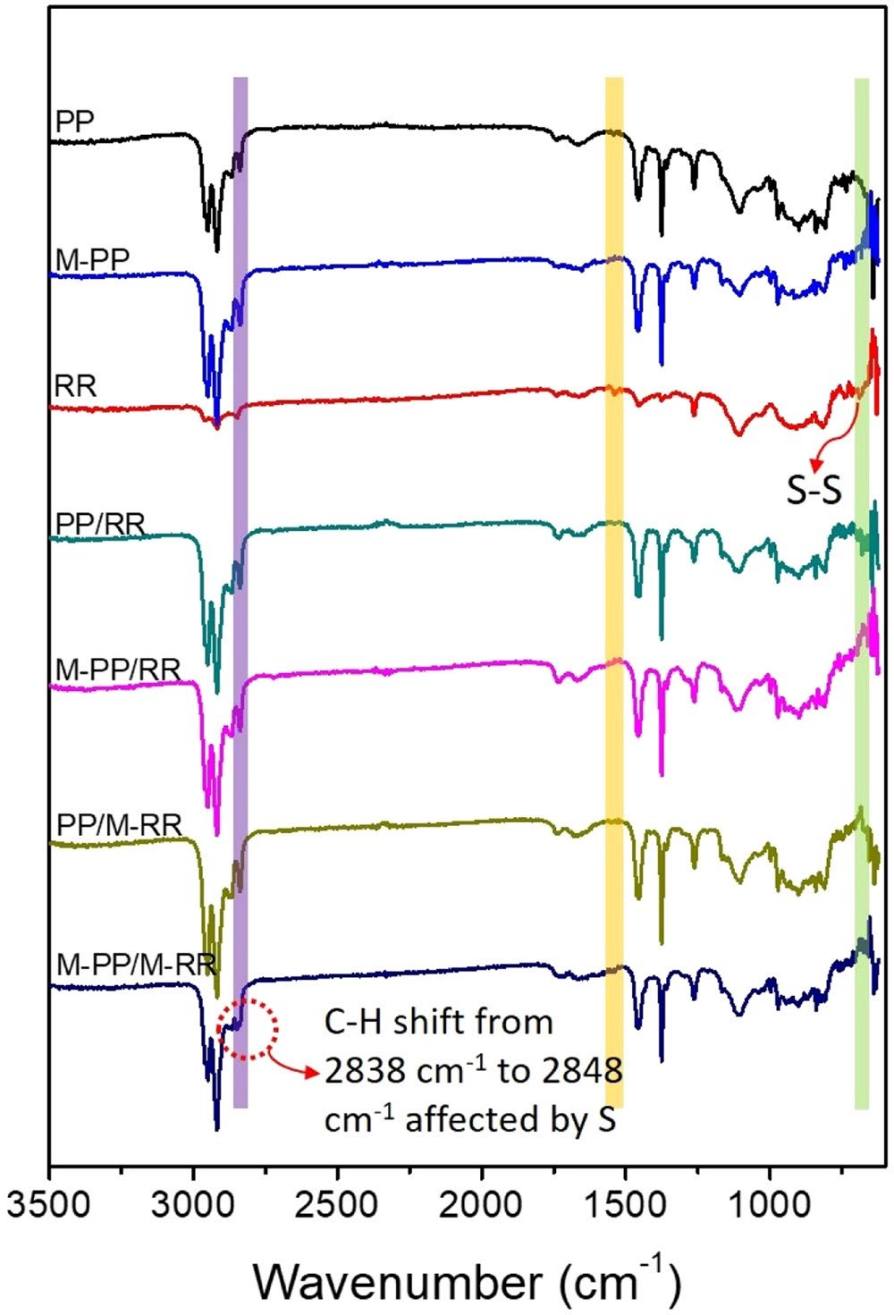
FT-IR spectra of PP, M-PP, RR, PP/RR, M-PP/RR, PP/M-RR and M-PP/M-RR.

To demonstrate the chemical reaction in the process of smelting, the Fourier transform infrared (FT-IR) spectra of different samples including polypropylene (PP), modified polypropylene (M-PP), recycled rubber (RR), binary blends of polypropylene and recycled rubber (PP/RR), binary blends of modified polypropylene and recycled rubber (M-PP/RR), binary blends of polypropylene and modified recycled rubber (PP/M-RR), and binary blends of modified polypropylene and modified recycled rubber (M-PP/M-RR) were carried out as shown in Fig. 1. The details of the preparation of the samples are shown in the experimental section. After blending, no obvious shift was observed at 2900 cm^−1^ and 2800 cm^−1^, which correspond to saturated stretch asymmetric vibration νas(-CH_3_) and symmetric vibration νs(>CH_2_) of the backbone, indicating that the majority of C-H moieties remain during processing. Notably, the spectrum of M-PP exhibited ν(COO) bands at 1700 cm^−1^, and when M-PP reacted with M-RR, this peak blue shifted slightly. In the meantime, peaks of C-H bonds from 2800 cm^−1^ to 2700 cm^−1^ became weaker after modification. As the stretching vibration peaks of S-H bond, S-C bond and S-S bond are very weak and hard to recognize. The IR spectra were carried out through total reflection mode. As showed in Fig. 1, the bending vibration of S-S in the fingerprint area disappeared after modifying and mechanical blending at high temperatures. Moreover, the C-H stretching vibration absorption peak of PP shift from 2838 cm^−1^ to 2848 cm^−1^ affected by S. The FT-IR spectra provided a possible mechanism, which is related to desulfuration and reaction occurred in a composite.

**Figure 2:**
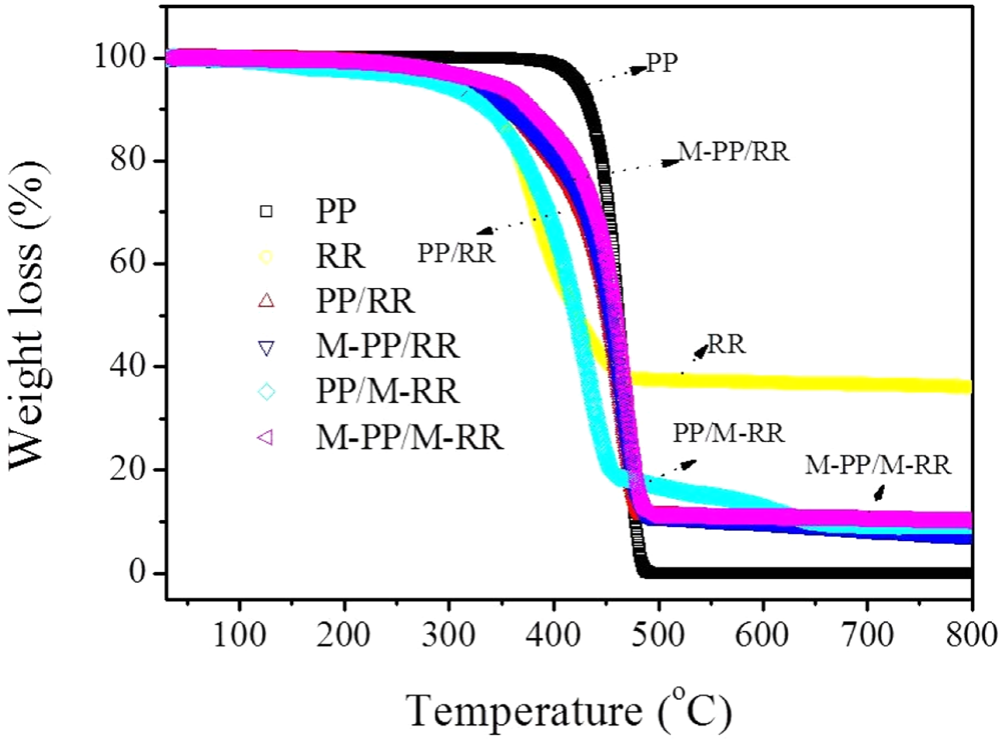
TGA curves of PP, RR, PP/RR, M-PP/RR, PP/M-RR, and M-PP/M-RR.

Thermal stability of PP, RR, PP/RR, M-PP/RR, PP/M-RR, and M-PP/M-RR was analyzed using TGA and summarized in Fig. 2. TGA curves of PP, RR, PP/RR, M-PP/RR, PP/M-RR, and M-PP/M-RR. The TGA curve of PP shows good thermal stability. No weight loss was observed before 400 °C. However, the thermal degradation profile for the RR sample exhibits weight loss steps at 300 °C. It is due to the degradation of the polymer and the decomposition of unstable additive chemicals. PP/RR, M-PP/RR, and M-PP/M-RR exhibit better thermal stability compared to that of RR. The TGA curve of PP/M-RR shows two-weight losses at 300 °C and 450 °C due to the release of unstable additive chemicals or the transformation of some sulfur-containing groups either by the reaction with desulfurizer. The weight loss observed at 450 °C is attributed to the decomposition of the PP non-crystal part twine with the rubber chain. The TGA curve obtained for M-PP/M-RR shows only one step of weight loss. The weight loss at about 400 °C is primarily due to the degradation of PP. The minor weight loss observed before 400 °C is presumably owing to the decomposition of small molecule accessory ingredient which is come from the RR after the desulfurization process. The result indicated that M-PP/M-RR sample exhibit better thermal stability than RR, PP/RR, M-PP/RR, and PP/M-RR possibly due to the good compatibility.

**Figure 3:**
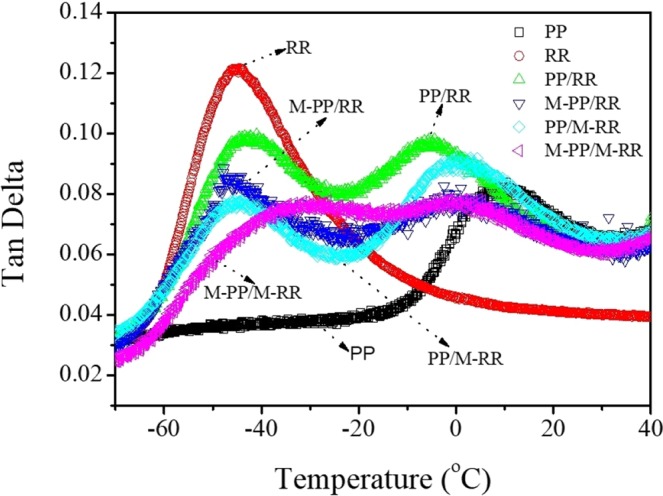
Tan δ curves of PP, RR, PP/RR, M-PP/RR, PP/M-RR, and M-PP/M-RR.

To further explore the compatibility of M-PP and M-RR, we carried out damping factor (tan δ) curves for PP, RR, PP/RR, M-PP/RR, PP/M-RR and M-PP/M-RR at a frequency of 1 Hz as shown in Fig. 3. Tan δ curves of PP, RR, PP/RR, M-PP/RR, PP/M-RR, and M-PP/M-RR. It shows that both PP and RR perform one primary thermal relaxation transition at −45 °C and 10 °C, respectively. A composite with stronger interfacial bonding tends to dissipate less energy and needs a higher temperature to initiate molecular motion, showing a lower tan δ magnitude and higher peak temperature than a composite with a poorly bonded interface^11^. The Fig. 3, the PP/RR, M-PP/RR, and PP/M-RR exhibited two independent tan δ peaks. Besides, the tan δ peaks of sample M-PP/M-RR were shifted closer comparing with each other, indicating the modification of PP and RR can enhance the inter solubility of molecules.

**Figure 4:**
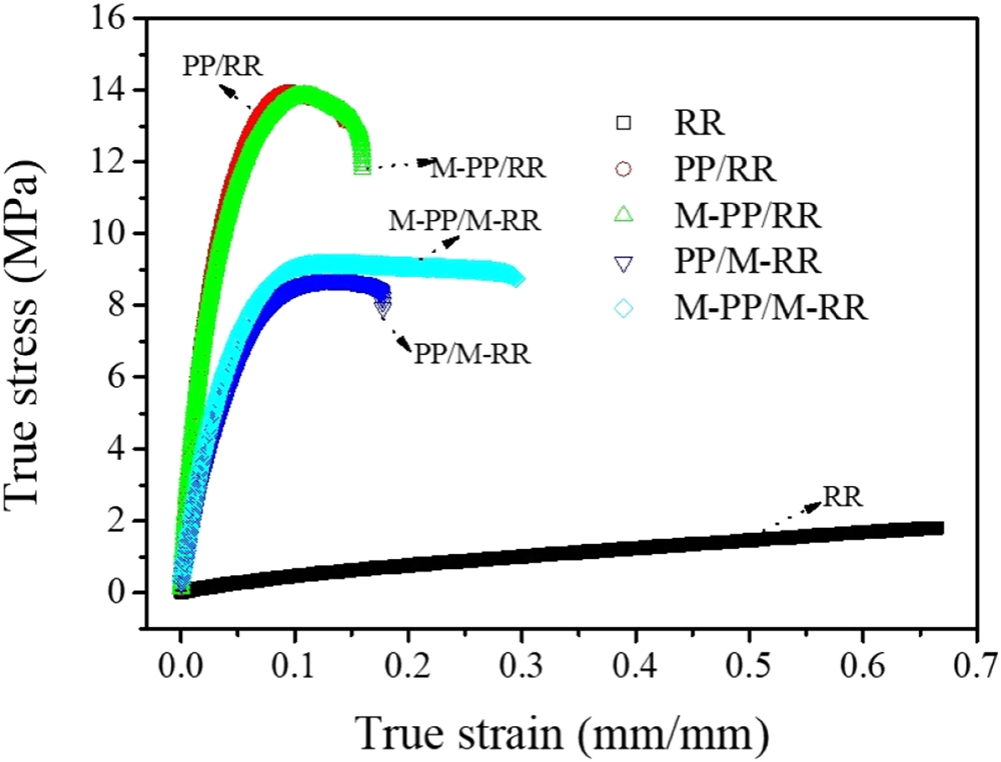
Tensile strength of RR, PP/RR, M-PP/RR, PP/M-RR, and M-PP/M-RR.

**Figure 5:**
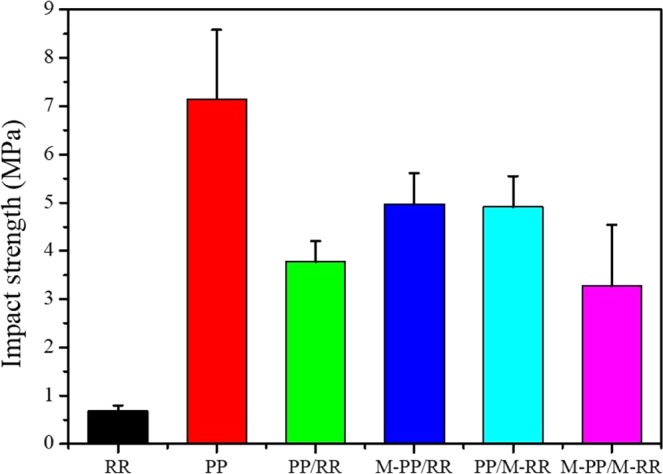
Impact strength of PP, RR, PP/RR, M-PP/RR, PP/M-RR, and M-PP/M-RR.

The tensile and impact strength of M-PP/M-RR, and the control blends are summarized in Figs. 4 and 5. As expected, it is measured that the desulfurated RR in PP or M-PP improves the malleability of the blends. The probable reason is that intermolecular forces and the rebinding chemical bond connection between PP and RR. It is indicated that the impact strength of the different blends is better in comparison with RR. While the elongation at break of the different blends become smaller than RR. Similar improvements in malleability with the incorporation of polymers have also been reported by previous researchers^12,13^. However, the tensile decreased by about 50% as compared to that of the neat PP. The tensile strength of RR increased after alloying can be expounded by the connection of PP and RR and by the morphological changes due to RR phase^14,15^. The improvement in impact strength can be explained by the effect of the desulfuration of RR on the consistency in the blend^16^. Similar improvements in the malleability of PP/EPR with the incorporation of EPR-g-MAH have been reported by the previous researches^17^. It is also worth noting that the impact strength of the M-PP/M-RR increased by about 440%, as compared to RR. Otherwise, the impact strength of the M-PP/M-RR is worse than that of PP. Due to rebinding connection of RR and PP of M-PP/M-RR forms a three-dimensional net structure, as a result of that brittle rupture compare to linear polymer PP.

**Figure 6:**
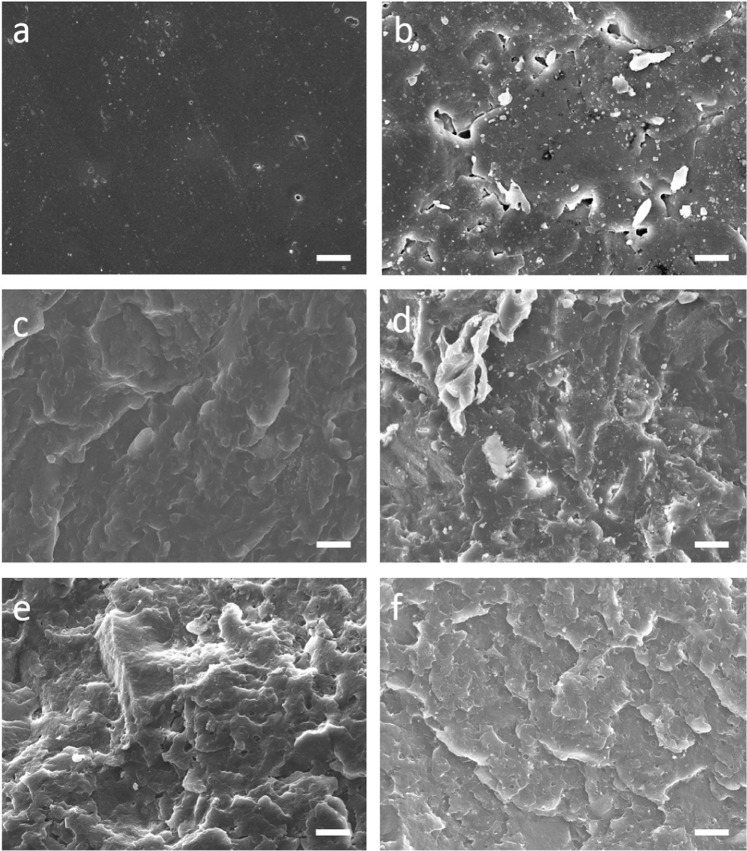
SEM images of (**a**) PP, (**b**) RR, (**c**) PP/RR, (**d**) M-PP/RR, (**e**) PP/M-RR and (**f**) M-PP/M-RR (Scale bar: 20 μm).

To further identify the microstructure of the composites, we carried out the SEM micrographs as shown in Fig. 6. SEM images of (a) PP, (b) RR, (c) PP/RR, (d) M-PP/RR, (e) PP/M-RR and (f) M-PP/M-RR. We can find that the micrograph of RR (Fig. 6b) shows bad machinability. In other words, the RR is not melted completely in a homogeneous manner. However, the presence of PP has promoted a better dispersion of the functionalized RR in the matrix. From the results of elongation at break, the elasticity became worse without modification.

Figure 6c–f shows the SEM micrographs of the compatibilized composites including PP/RR, M-PP/RR, PP/M-RR, and M-PP/M-RR. It is shown that the use of the M-PP has significantly promoted machinability of RR in the matrix which means that the composite prepared using M-PP shows a clearer increase of the intermolecular forces of the M-RR. This has been investigated by the good elongation at a break that was obtained with the former system. A fine and uniform dispersion of RR in the PP phase is observed (Fig. 6f). The increase of compatibility of the RR phase and PP phase is due to the decrease of the interfacial tension between the dispersed PP matrix and the RR phase.

## Conclusion

The nanocomposites of maleic anhydride-modified polypropylene/desulfuration modified recycled rubber (M-PP/M-RR) compare with different unmodified binary blends were investigated to confirm their chemical construction impact on the thermostability, mechanical strength, and morphological properties. The incorporation of PP as well as M-PP into the RR matrix led to a significant improvement in the thermal stability. Besides, the M-PP/M-RR nanocomposites showed a significant improvement in the mechanical properties compared to RR. This is due to the enhancement of interpenetration and interfacial bonding between PP and RR. SEM micrographs illustrate that RR has a nodular microstructure. The modification of RR promotes a better dispersion of desulfurated RR in the polymer matrix. Future expectations can improve rubber recycling and reuse, reduce the cost of raw materials in the rubber industry, and used in automotive rubber materials or electronic product casings, etc.
